# Impact of diagnostic stewardship interventions in the collection process of fungal blood cultures

**DOI:** 10.1017/ice.2023.182

**Published:** 2024-03

**Authors:** Miguel A. Chavez, Satish Munigala, Carey-Ann D. Burnham, Melanie L. Yarbrough, Crystal Squires, Josephine Fox, Heather Gasama, Kevin Hsueh, David K. Warren

**Affiliations:** 1 Division of Infectious Diseases, Department of Medicine, Washington University School of Medicine, St. Louis, Missouri; 2 Department of Pathology & Immunology, Washington University School of Medicine, St. Louis, Missouri; 3 Department of Laboratories, Barnes-Jewish Hospital, St. Louis, Missouri; 4 Department of Hospital Epidemiology and Infection Prevention, Barnes Jewish Hospital, St. Louis, Missouri

## Abstract

We implemented 2 interventions to improve utilization and contamination at our institution: kits to improve appropriate sample collection and an electronic order alert displaying appropriate indications of fungal blood cultures. An electronic order alert when ordering fungal blood cultures was associated with decreased utilization without decrease in positivity rate.

Fungal blood cultures (FBCs) are used for the diagnosis of some invasive fungal infections such as *Fusarium* spp and *Histoplasma capsulatum* infections.^1^ However, these tests take longer compared to other fungal diagnostics (eg, *Histoplasma* urine antigen)^2^; they may not be warranted, particularly to detect candidemia, the most common cause of fungemia^3^; and they may have little utility in certain populations.^4^ There are no current guidelines regarding the specific indications for performing an FBC, which can result in overuse and low diagnostic yield.^5^ We observed an incidental increase in bacterial recovery from FBC at our institution and subsequently evaluated 2 interventions to improve FBC utilization.

## Methods

### Setting and study design

We performed a retrospective study between June 2018 and March 2023 of hospitalized adults who had fungal blood cultures and routine blood cultures performed at Barnes-Jewish Hospital (BJH), a 1,250-bed academic hospital in St. Louis, Missouri, where Epic software (Epic Systems, Verona, WI) is used for the electronic medical record (EMR) system.

### Intervention design and implementation

During the study period, we implemented 2 hospital-wide interventions. In the first intervention, we aimed to improve FBC collection technique. On April 16, 2021, kits were created with instructions on the supplies needed and appropriate technique for sample collection, including isolator tube disinfection (intervention 1) (Supplementary Material online). These kits were followed by education of nursing leaders from each of our hospital units. The second intervention was implemented on October 13, 2021, and consisted of an electronic order alert displaying the following: “Filamentous fungal blood cultures should only be ordered in patients with suspected disseminated infection due to mold or dimorphic fungi such as Histoplasma and Blastomyces”^6^ (intervention 2) (Supplementary Material online).

### Blood cultures

FBCs were collected using the Wampole Isolator System (Wampole Laboratories, Cranbury, NJ): 10 mL blood was collected in the fungal isolator tube and then transported to the laboratory. Tubes were centrifuged according to the manufacturer’s instructions; supernatant was discarded; and the concentrate was plated to chocolate, brain heart infusion (BHI), and Sabouraud dextrose agars (SDA). Chocolate plates were incubated aerobically at 35°C and discarded after 5 days if no growth occurred. BHI and SDA were incubated aerobically at 30°C and examined daily for 7 days and then twice weekly for a total incubation time of 28 days. Yeasts were identified using matrix-assisted laser desorption ionization-time of flight mass spectrometry (MALDI-TOF MS; Bruker BioTyper, Bruker Daltonics, Billerica, MA). Filamentous molds were identified using a combination of macroscopic and microscopic characteristics and sequence-based methods performed at a reference laboratory. Organisms were reported to the EMR based upon expert review (eg, plates rounds) considering organism isolated, timing of growth, organism location on the plate, etc.

### Study outcomes and statistical analysis

Our primary end points were FBC utilization, proportion of positive FBC, and rate of skin commensal recovery reported in the EMR. Skin commensal organisms included those in the National Health Safety Network (NHSN) organism list.^7^ Continuous variables were expressed as median with interquartile range, as appropriate, and assessed using the Mann-Whitney *U* test. Categorical variables were presented as absolute numbers and frequencies and were compared using the χ^2^ test or the Fisher exact test, as appropriate. Two-sided *P* < .05 was considered statistically significant. Analyses were performed with STATA version 16.1 software (StataCorp, College Station, TX).

## Results

**Figure 1.**
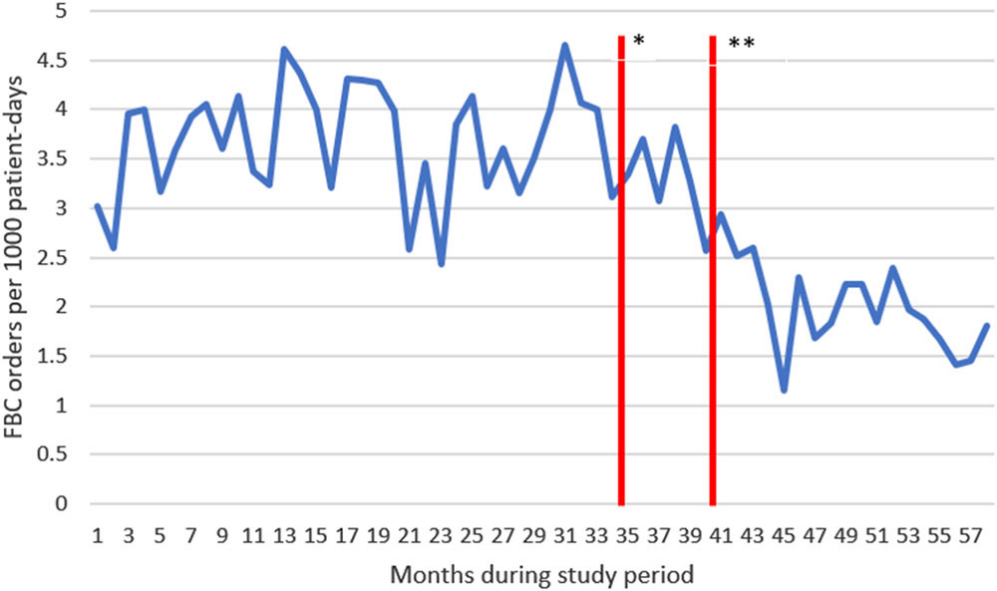
Frequency of fungal blood culture performed during the study period (June 2018–March 2023). Note. BJH, Barnes Jewish Hospital; FBC, fungal blood cultures. *Intervention 1 was implemented on April 16, 2021. **Intervention 2 was implemented on October 13, 2021.

In total, 5,140 FBCs were performed during 4,250 admissions: 3,511 during 2,834 admissions in preintervention period 1, 543 during 466 admissions in postintervention period 1, and 1,086 during 950 admissions in postintervention period 2 (Fig. 1). The FBC positivity rate was 6.5%: 6.4% preintervention 1, 6.3% postintervention 1, and 7.0% postintervention 2 (*P* = .73). We did not see a difference after intervention 1 or after intervention 2 (Table 1). *Candida* spp were the fungi most commonly isolated from FBCs (78 of 150), followed by *Histoplasma* spp (23 of 150), and *Cryptococcus* spp (17 of 150). We observed an increase in recovery rate of *Histoplasma* spp during postintervention period 2 but not of *Candida* spp or *Cryptococcus* spp (Table 1). The FBC skin commensal recovery rate was not different in postintervention period 1: 0.7% before implementation versus 0.7% after implementation 1 (*P* = .934). FBC utilization significantly decreased in postintervention period 2: 3.3 FBC performed per 1,000 patient days versus 1.9 per 1,000 patient days after intervention 2 (*P* < .001).

**Table 1.** Pathogens Isolated From Fungal Blood Cultures Before and After Intervention 1

Microorganisms, n (%)	Preintervention Period 1 (n=3,511)^a^	Postintervention Period 1 (n=1,629)^b^	*P* Value	Preintervention Period 2 (n=4,054)^c^	Postintervention Period 2 (n=1,086)^d^	*P* Value
Positive fungal blood culture	223 (6.4)	110 (6.7)	.587	257 (6.3)	76 (7)	.433
Fungi	100 (2.9)	50 (3.1)	.661	116 (2.9)	34 (3.1)	.64
*Candida* spp	53 (1.5)	23 (1.4)	.787	64 (1.6)	14 (1.8)	.56
*Histoplasma* spp	10 (0.3)	13 (0.8)	.01	12 (0.3)	11 (1)	.002
*Cryptococcus* spp	12 (0.3)	5 (0.3)	.84	12 (0.3)	5 (0.5)	.402
**Other**						
Bacterial pathogens	124 (3.5)	55 (3.4)	.777	142 (3.5)	37 (3.4)	.879
Skin commensals^e^	23 (0.7)	11 (0.7)	.934	27 (0.7)	7 (0.6)	.938

a Preintervention period 1: June 1, 2018, to April 15, 2021.

b Postintervention period 1: April 16, 2021, to March 31, 2023.

c Preintervention period 2: June 1, 2018, to October 12, 2021.

d Postintervention period 2: October 13, 2021, to March 31, 2023.

e Skin commensal organisms included coagulase-negative staphylococci (CoNS), *Bacillus* spp, and *viridans* group streptococci.

## Discussion

A collection kit with instructions on appropriate collection technique had no effect on FBC recovery of skin commensals, whereas an electronic order alert displaying appropriate indications for FBC was associated with decreased FBC utilization without changes in positivity rate except an increase in *Histoplasma* spp isolation.

The fungal isolator tube has historically been reserved for patients with sepsis suspected from endemic mycoses and filamentous fungi that would not usually be recovered by routine blood cultures.^6^ Current modern blood-culture systems can effectively detect candidemia, which precludes the use of dedicated FBC for this purpose.^3^ Furthermore, routine blood cultures can also be useful for detection of some invasive mold infections, such as fusariosis and scedosporiosis, but not for other molds.^3^ One study demonstrated poor understanding of the utility of another fungal diagnostic (β-D-glucan) by nonexperts, which resulted in half of orders being inappropriate.^8^ In our study, a simple intervention of displaying appropriate indications for FBC decreased ordering by 36%, suggesting that proper design of electronic order sets with education can reduce excessive ordering of FBC without affecting the overall positivity. Nevertheless, future studies should evaluate the clinical impact of these diagnostic stewardship interventions.

We observed an increased isolation of *Histoplasma* spp, likely due to fewer inappropriate FBC obtained during postintervention period 2. Although fungemia may not be associated with worse outcomes, detection of fungemia can possibly cause a more aggressive treatment and closer follow-up than nonfungemic patients resulting in similar survival.^9^ Future studies should evaluate whether positive FBC provided any clinical benefit in addition to other routine fungal diagnostic testing, such as *Histoplasma* urine antigen, which usually return results sooner than FBCs.^2^

Our study had several limitations. We used a retrospective design, and we did not conduct a chart review of patient characteristics and clinical outcomes of all patients with FBC. In addition, we were not able to assess plate contamination by environmental or skin organisms that was not reported to the EMR. However, we did examine the reported isolation of skin commensal bacteria from FBCs, which may result in increased work-up in the microbiology laboratory and unnecessary additional treatment of patients. Also, we were not able to evaluate the reasons why the kit was not successful, but we believe the potential benefit may have been marginal to see any significant change.

This is the first study to evaluate an order-entry intervention to improve FBC utilization that could easily be replicated at other institutions. Reducing unnecessary diagnostic testing of hospitalized patients is important for reducing overall healthcare costs and improving care.
